# Audio Interference Suppressor in Analog Audio Interface

**DOI:** 10.3390/s25185868

**Published:** 2025-09-19

**Authors:** Vladimir Olujić, Siniša Fajt, Vlado Sruk, Miljenko Krhen

**Affiliations:** 1 University of Zagreb Faculty of Electrical Engineering and Computing, Unska 3, 10000 Zagreb, Croatia; vladimir.olujic2@fer.unizg.hr (V.O.); sinisa.fajt@fer.unizg.hr (S.F.); 2 University of Zagreb Faculty of Textile Technology, Prilaz baruna Filipovića 28a, 10000 Zagreb, Croatia; miljenko.krhen@ttf.unizg.hr

**Keywords:** audio interference, EMI suppression, ground loops, unbalanced interconnects, analog audio systems, AISAAI suppressor

## Abstract

Audio systems with unbalanced connections are susceptible to interference from ground loops, which manifests as hum and noise. This paper introduces and evaluates a novel passive Audio Interference Suppressor in Analog Audio Interface (AISAAI) designed to mitigate this problem. The AISAAI circuit is inserted between an audio device’s rectifier ground and its protective earth terminal, creating an optimized impedance path that reduces interference while ensuring safety. This approach is analyzed within a proposed Analog Audio Interconnection System (AAIS) framework. Experimental results show that common-mode voltages from protective earth potential differences are the primary source of interference. The optimized AISAAI suppressor achieves a consistent 15–30 dB reduction in measured audio interference across the audio band, regardless of the interconnect cable characteristics. This study confirms AISAAI as an effective solution for reducing ground loop noise in unbalanced audio systems and underlines the usefulness of the AAIS model for systemic analysis.

## 1. Introduction

To achieve accurate reproduction of audio signals in audio systems, the integrity of the signal must be guaranteed throughout the transmission path from the source to the receiver. However, these systems are inherently susceptible to audio interference. Audio interference arises from the intrusion of noise, hum and hiss originating from both external and internal sources through various electromagnetic, galvanic, capacitive and inductive mechanisms [1,2,3,4], as well as their superposition with the desired audio signal and the resulting nonlinear distortions. In addition to interference and noise, these distortions appear as unwanted hum, especially at 50 Hz and its harmonics. Such interference can obscure intricate signal details and significantly degrade the perceived sound quality [2,5,6].

A major challenge is the occurrence of audio interference due to ground loops and galvanic connections, a phenomenon that is particularly prevalent in systems with multiple devices connected via unbalanced interconnect cables [2,7]. The potential differences that arise in the protective earth conductors (PE) of the power supply cables in such parasitically generated loops induce a parasitic current. This current subsequently induces a voltage drop across the impedance of the negative conductor and is superimposed on the audio signal according to the principle of superposition on a common impedance and thus becomes a predominant source of interference.

In practice, audiophiles and experts often observe audible deviations when using different connection cables. However, these deviations cannot usually be fully explained or quantified by objective standard evaluations of the cables as discrete components. This incongruity is also reflected in the scientific literature. While some studies [8] conclude that the influence of cables is minimal based on laboratory evaluations, other studies [9] confirm that interconnect cables influence sound, but acknowledge that it is difficult to determine all physical causes by measurements on the cables alone [9,10]. It is obvious that the evaluation of cables detached from the overall system context and the existing audio interference is not sufficient for a comprehensive understanding of the problem. Effective mitigation of audio interference, taking into account the interactions within the system, is essential for transparent reproduction. Current grounding and filtering methods [1,2,3,11] do not always solve the problem satisfactorily, especially in complex systems characterized by unavoidable ground potential differences.

Several established methods exist to mitigate audio interference from ground loops, though each presents distinct limitations. The primary professional solution involves audio isolation transformers, which break the ground loop through galvanic isolation. While effective, this approach can compromise high-fidelity signals by introducing frequency response limitations, phase distortion, and significant cost [12,13]. Furthermore, even high-quality transformers can introduce signal degradation, such as restricted frequency response or phase distortion, and represent a costly solution for high-fidelity applications.

Filtering techniques, such as common-mode chokes, offer another approach. However, their efficacy is often limited against the low-frequency hum (50/60 Hz and its harmonics) that is the principal component of ground loop interference [1]. Such methods attempt to treat a symptom (the noise) rather than the root cause (the ground loop itself).

A widely seen but fundamentally unsafe practice is “ground lifting”, where the PE conductor is deliberately disconnected using a 3-to-2 prong adapter. This action eliminates the ground loop at the cost of defeating the system’s safety mechanism, creating a serious risk of electric shock for the user [14].

Collectively, the shortcomings of existing methods—ranging from compromised audio fidelity to severe safety hazards—highlight a clear need for a solution that is simultaneously passive, safe, cost-effective, and capable of suppressing ground loop interference without degrading the audio signal [1,14,15,16]. This paper addresses this need at a systemic level and offers two important contributions. First, the concept of Analog Audio Interconnection System (AAIS) is introduced as an analytical framework that includes the output audio interface of the source, the connecting cable and the input audio interface of the receiver as a unified entity. This approach enables a better understanding of the interactions between the components and the influence of interference in the actual signal path. A key innovation of the Audio Interference Suppressor in Analog Audio Interface (AISAAI) is its unique placement: unlike traditional external isolators that are placed in the signal path between devices, the AISAAI is a circuit designed for integration inside the audio device itself. Secondly, and this is the main technical contribution, the paper presents the design, analysis and validation of a novel solution called AISAAI. The AISAAI is a circuit inserted between the rectifier ground point of the audio device and its protective ground point to suppress audio interference. Its operating principle is based on changing the impedance of this connection through an optimized combination of passive, linear and non-linear components while ensuring a safety function in the event of a fault.

This article is structured as follows: After this introduction, the next section presents the results of an objective characterization of the basic electrical parameters (R, L, C) of various commercially available unbalanced audio cables. The third section introduces the AAIS model and presents an experimental analysis of the influence of the most important sources of audio interference—the configuration of the mains supply and the termination of the ground loop. The fourth section describes in detail the development of the AISAAI suppressor, including the construction of a laboratory setup for testing and analyzing the effectiveness of its individual components. The fifth section explains the selection of the final optimal AISAAI circuit configuration, explains the operating principle and safety aspects, and presents the results of experimental validation under realistic conditions with different cables. The paper concludes with a discussion of the results obtained and a conclusion.

## 2. Audio Cable Measurement

To quantify the fundamental electrical differences between commercially available interconnect cables that may contribute to performance differences in audio systems, laboratory measurements of the basic RLC parameters were performed. The measurements were performed on a sample of twelve different unbalanced audio cables, without the direct influence of the source and receiver interfaces. The measured cables came from different manufacturers, designs and technical versions as well as from different price ranges.

Although several parameters of the conductors and connectors were considered, the analysis in this section focuses on the characteristic results for resistance (R), inductance (L) and capacitance (C) per unit length (1 m). These quantities represent the basic parameters that define the electrical behavior of a cable. To improve clarity, the results of the cable measurements are summarized in Table 1. The table reports the minimum, median, and maximum values of capacitance, inductance, and resistance across the twelve commercially available audio cables, along with representative examples identified by manufacturer and model. This work highlights the significant variation in electrical properties and provides a straightforward basis for comparison.

These results clearly illustrate the wide dispersion of electrical parameters among commercial cables, underlining their significant influence on susceptibility to interference and justifying the need for a systematic suppressor solution. These deviations in the parameters are partly due to different design approaches. In standard unbalanced audio cables, the negative conductor for the audio signal is usually a conductor corresponding to the positive, and the cable is shielded from external Electromagnetic Interference (EMI) by a separate conductive shield (often in a coaxial configuration). Shield connection strategies vary between manufacturers, sometimes involving grounding at the lower impedance end, at both ends, or through an RC network. These design differences significantly affect the impedance of the negative conductor and, consequently, the cable’s EMI behavior.

Measuring the capacitance between the signal (+) and return (−) conductors revealed significant differences of up to two orders of magnitude between the cables tested. The lowest measured value in the group was 38.72 pF/m (e.g., Shunyata Research Aries cable, Shunyata Research, Poulsbo, WA, USA), a value approaching the median was 111.35 pF/m (AudioQuest Amazon, AudioQuest, Irvine, CA, USA), while the highest measured value reached 1252.53 pF/m (Transparent Reference, Transparent Audio, Saco, ME, USA).

There were also differences of two orders of magnitude when measuring the conductor inductance. The minimum measured inductance of the (negative) return conductor was 0.27 μH/m (Accuphase Super Refined cable, Accuphase Laboratory, Inc., Yokohama, Japan), the approximate mean value was 3.63 μH/m (Transparent MusicLink Ultra, Transparent Audio, Saco, ME, USA), and the maximum value was recorded for the Transparent Reference cable at 27.40 μH/m.

When measuring the resistance of the return conductor, which serves as the signal earthing path, the measured values between the different versions showed differences of an order of magnitude. The minimum measured resistance of the negative conductor was 5.54 mΩ/m (Accuphase Super Refined Cable), a value of 16.71 mΩ/m (Transparent Reference) corresponded approximately to the median, while the maximum value of 43.41 mΩ/m was measured with an unbranded, generic audio cable.

In simpler, often non-branded designs, a coaxial construction is used where the cable shield itself performs a dual function: It serves both as a return (negative) path for the audio signal and as a shield against external EMI. In this case, the shield of the coaxial cable, which acts as a negative conductor, is necessarily connected at both ends of the cable. Different designs and connection methods between the shield and the negative conductor directly affect the impedance of the signal return path, which is critical to the behavior of the cable in the presence of EMI.

Based on the measurements of the RLC parameters and the large differences found, especially in the impedance of the negative conductor, two specific cables with significantly different characteristics were selected for further detailed investigation of the influence of the cable on signal transmission and susceptibility to EMI within the system. The selected cables are the Accuphase Super Refined Cable (ASRC for short), which is characterized by a low negative conductor resistance of 5.54 mΩ/m, a negative conductor inductance of 1.04 μH/m and a capacitance between conductors of 378.6 pF/m, and an unbranded generic audio cable (NBAC for short) with a negative conductor resistance of 90.7 mΩ/m, a negative conductor inductance of 1.65 μH/m and a capacitance of 112.7 pF/m.

The parasitic capacitance between the positive and negative conductors of an audio cable of the order of a hundred or several hundred pF/m, together with the parasitic input capacitance of the receiver (of the order of a hundred pF), affects the linear distortion of the audio system, in particular the amplitude response, which is typically outside the audible range (i.e., in the range of several hundred kHz and higher). This is due to the generally very low output impedance of the source, which is regularly only a few tens of Ω for active semiconductor circuits.

Possible problems with resonant frequencies in audio cables under certain conditions of high total parasitic capacitance in the audio signal path combined with an extremely low output impedance of the source are very specific cases and not the subject of this discussion. Furthermore, the parasitic capacitance of the audio cable is not an integral part of the observed parasitic loop of the analog audio interconnect system.

The measured parameters were recalculated for a cable length of 1 m. Since much longer interconnect cables are often used in practice (e.g., for connecting mono power amplifiers), the total impedance of the negative conductor can increase significantly at such lengths, potentially exacerbating EMI problems. The relationship between conductor impedance and possible resonance effects as a function of frequency is discussed in [17].

To illustrate the effects of frequency on the total impedance of the negative conductor for the selected cables, Table 2 shows the calculated increase in inductive reactance and impedance modulus for the 1 m ASRC and NBAC cables. The table also shows the relative contribution of the inductive component (the X_L_/R and ∣Z∣/R ratio) and a comparative ratio of the impedances of these two cables at different frequencies, which clearly shows how their behavior differs with increasing frequency.

For the selected ASRC and NBAC cables, it can be clearly seen that the total impedance of the return conductor, ∣Z∣, increases significantly with frequency for both cables, depending on the frequency in the range from 100 Hz to 100 kHz. This increase is primarily due to the influence of the conductor’s inductance, whose reactance increases linearly with the frequency. The analysis of the X_L_/R and |Z|/R ratios confirms that the inductive component becomes the dominant factor in the total impedance at frequencies of only a few kHz, especially for the ASRC cable, which has a very low DC resistance in the return path.

The comparison of the two cables, which is determined by the ratio of their impedances (Z_ASRC_/Z_NBAC_ ratio), quantitatively confirms the significant differences in their characteristics. The Accuphase Super Refined Cable (ASRC) has a consistently lower return path impedance than the unbranded cable (NBAC) over the entire observed frequency range. At 10 kHz, for example, the return path impedance of the ASRC cable is about 48% of the impedance of the NBAC cable, while at 100 kHz this ratio is about 63%. These results emphasize the importance of cable construction to the return path impedance characteristics, which is critical to understanding the interaction of audio cables with EMI.

## 3. The AAIS Model: Structural and Functional Analysis

The subjectively perceived deterioration in the quality of the transmitted audio signal, such as the change in timbre when using different connection cables, is a phenomenon that requires analysis in the wider context of the entire audio system and its operating environment. Objective measurements and analysis show that the final result is significantly influenced not only by the characteristics of the audio cable itself (construction, materials, R, L, C parameters), but also by other factors. These include the technological design and electrical parameters of the output audio interface (as the output stage of the audio signal source) and the input audio interface (as the input stage of the audio signal receiver), audio interference from the power grid, grounding effects (e.g., parasitic currents through the PE of the power supply cables for audio equipment and their technological design) and finally EMI in the environment of the audio system.

The AAIS model, as defined in the Introduction, enables an accurate analysis of the interactions between the key components of analog audio systems and their impact on the quality of signal transmission. The AAIS is defined as a comprehensive system that includes the output interface of the analog audio signal source (device AD_1_), the audio interconnect cable, the input interface of the analog audio signal receiver (device AD_2_), the power supply cable for the source (device AD_1_), and the power supply cable for the receiver (device AD_2_). In this paper, the AAIS is considered a basic subsystem within a comprehensive audio system model that allows a systematic understanding of the signal transmission path and the identification of its degradation mechanisms.

The concept of an audio interface in the strict sense, which aims to suppress EMI by interpolating an audio transformer into the parasitic loop circuit, is mentioned in Whitlock’s work [7,12,17]. The term AAIS is further elaborated in the context of its affiliation with the broader concept of interconnection of analog audio devices, which includes both the output and input points of the system. This approach facilitates a detailed characterization of the audio signal behavior within the system and the analysis of audio interference under real-world conditions. As part of the proposed model, a special interference suppression circuit AISAAI is introduced. This circuit is integrated between the connection point of the protective ground of the audio device (MPCPE) and the Power Ground (PG) of the rectifier.

**Figure 1:**
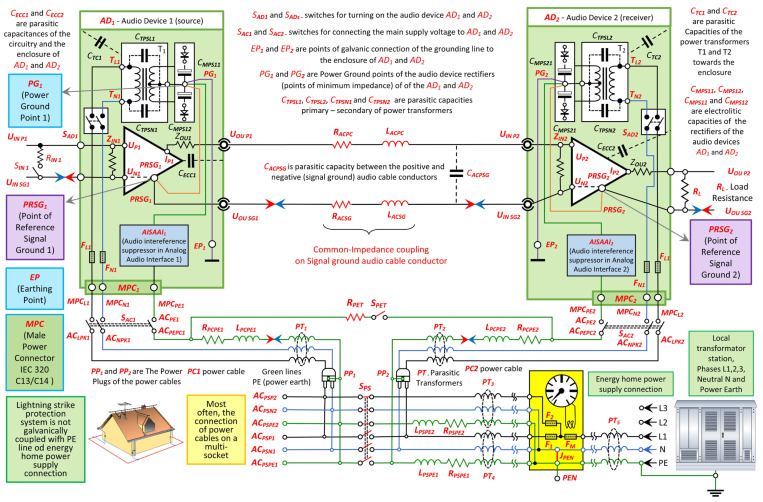
Block diagram of the comprehensive audio system model.

The block diagram of the comprehensive audio system model is shown in Figure 1. It illustrates the functional relationships between the analog output and input audio interfaces (AAI) and the position of the AISAAI within the system. The diagram includes the signal source (AD_1_—preamplifier), the receiver (AD_2_—power amplifier), the connecting cable and the respective power supply cables (PC_1_ and PC_2_). Such a configuration allows a thorough analysis of the interactions between all major components and an evaluation of the effectiveness of the AISAAI circuit in reducing audio interference and improving the quality of analog audio signal transmission. A detailed description of the function and implementation of the AISAAI element is given later in this paper.

Based on the block diagram of the comprehensive audio system model, a corresponding block diagram of the AAIS can be derived. This concept was previously introduced with a description of its components. Audio interference, which is largely caused by unavoidable ground potential differences, is superimposed on the transmitted wanted signal as a voltage drop across the return conductor of the connecting cable, more precisely by superimposition with its common impedance, which is shared by both the wanted signal and the interference signal.

The block diagram in Figure 1 shows a galvanic connection of the PE conductor. The AD_1_ device is connected via its power supply cable to the MPCPE_1_ point of the MPC_1_ mains plug (typically a type IEC 320 C13/C14) on the device chassis and then internally galvanically connected to the PG_1_ point [4,18]. From this point, there is a connection to the electrically conductive shielding or to the chassis of the device at point EP_1_ and to the reference signal grounding point PRSG_1_. Identical galvanic connections, as described, also exist within the device AD_2_. When two audio devices, AD_1_ and AD_2_, are connected with an unbalanced audio cable to transmit the wanted signal from the source AD_1_ to the receiver AD_2_, a parasitic loop is created simultaneously. This loop is created by the galvanic connection of the negative conductor of the audio cable between the previously defined protective earthing networks, or more precisely, their signal grounds. While illustrated for a two-device setup, the suppressor concept is equally applicable to more complex audio chains with multiple interconnected devices or longer cable runs, where ground loop interference can be even more pronounced.

**Figure 2:**
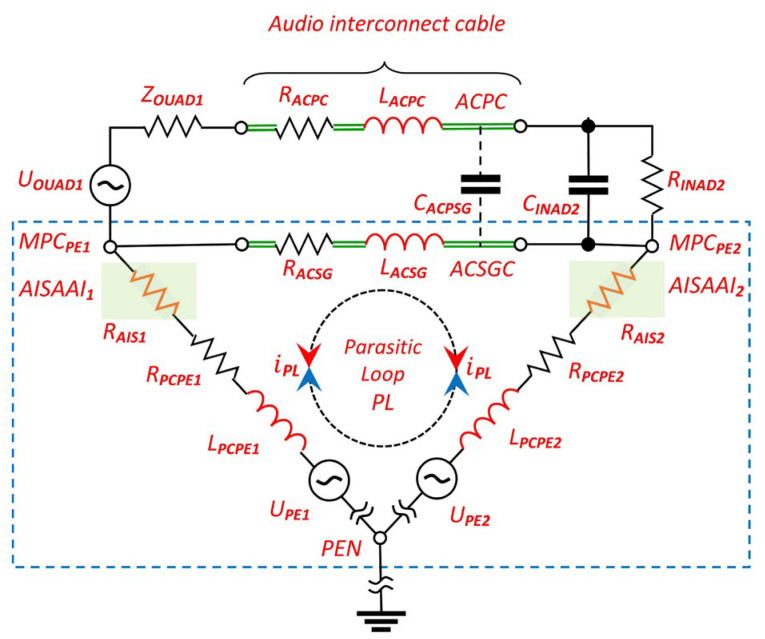
Equivalent block diagram of the Analog Audio Interconnection System (AAIS).

According to Figure 2, the occurrence of parasitic current in the loop formed by the points PEN, MPCPE_1_ and MPCPE_2_ is triggered by voltages of the symbolic generators U_PE1_ and U_PE2_. These voltages are the result of potential differences caused by the induction of external EMI in the PE conductors or by the action of parasitic transformers between the L, N and PE conductors in the power supply cables PC_1_ and PC_2_ of the audio devices AD_1_ and AD_2_.

The parasitic loop is indicated by the blue dashed quadrant. It is taken into account when analyzing the effects of the superposition of audio interference on the useful signal and when creating the vector diagram. The positive conductor (+) of the audio cable has a negligible effect on the parasitic current flow within the loop, as the input resistance R_INAD2_ is about 47 kΩ, which is a common value for unbalanced input stages of audio devices. The parasitic capacitances of the audio cable (C_ACPSG_) and the input stage of the receiver (C_INAD2_) are low and are in the order of 100 pF. In conjunction with the low output resistance of the AD_1_ source, their influence on the amplitude response is therefore clearly outside the audio frequency range.

Crucially, the diagram illustrates the unique placement of the AISAAI blocks. They are shown integrated within the grounding structure of each device’s interface—connecting the rectifier ground point to the PE terminal—a topology that is fundamentally different from traditional external isolators placed in the direct signal path.

It is critical to note that the AISAAI suppressor operates entirely outside the direct audio signal path, only modifying the impedance of the ground reference connection. This topology ensures that the suppressor does not introduce frequency-dependent phase shift or amplitude distortion to the transmitted audio signal, thereby preserving its integrity.

Based on the comprehensive model of the audio system, it can be determined that the AAIS consists of the following components:

- AAIS -> Analog Audio Interconnection System;
- AISAAI -> Audio Interference Suppressor in Analog Audio Interface;
- R_AIS_ -> Operating resistance within the AISAAI circuit;
- ACSGC -> Audio Cable Signal Ground Conductor;
- ACPC -> Audio Cable Positive Conductor;
- PL -> Parasitic Loop;
- i_PL_ -> Parasitic Loop Current;
- U_OUAD1_ -> The voltage of the electromotive force of the source AD_1_;
- Z_OUAD1_ -> Output impedance of the source (audio device AD_1_);
- C_INAD2_ -> Input (parasitic) capacitance of audio device AD_2_ ≈ 100 pF;
- R_INAD2_ -> Input resistance of audio device AD_2_ ≈ 47 kΩ;
- R_ACSG_ -> Resistance of the (−) negative conductor (Signal Ground) of the unbalanced audio cable;
- L_ACSG_ -> Inductance of the (−) negative conductor (Signal Ground) of the unbalanced audio cable;
- R_ACPC_ -> Resistance of the (+) positive conductor of the unbalanced audio cable;
- L_ACPC_ -> Inductance of the (+) positive conductor of the unbalanced audio cable;
- C_ACPSG_ -> Parasitic capacitance of the audio cable between the (+) and (−) conductors;
- R_PCPE1_ and R_PCPE2_ -> Resistances of the PE conductors in power cables PC_1_ and PC_2_ for supplying AD_1_ and AD_2_;
- L_PCPE1_ and L_PCPE2_ -> Inductances of the PE conductors in power cables PC_1_ and PC_2_ for supplying AD_1_ and AD_2_;
- U_PE1_ and U_PE2_ -> Voltage of EMI on the PE conductors in power cables PC_1_ and PC_2_ for supplying AD_1_ and AD_2_;
- MPCPE_1_ and MPCPE_2_ -> Connection points of the PE conductor of the power supply cable for the audio devices;
- PEN -> Common connection point of the PE conductors of the power distribution network cables.

## 4. Analysis of Audio Interference and Proposal of the AISAAI Suppressor

During the analysis of the system operation under real conditions, the measurements confirmed the presence of audio interferences that affect the quality of sound reproduction, especially within the analog audio interface. The measurements showed that the characteristics of the audio cable as an isolated component have no direct influence on the penetration of hum, interference and noise into the audio system. However, considering the interference emanating from the audio equipment environment, the need to develop additional circuits to suppress audio interference in audio lines emerged [3,4,17,19,20]. A discussion on the degree of EMI suppression by interpolating an audio transformer into the audio interface loop based on the quality or technological design of the audio transformer is addressed in the work [12,21].

The proposed AISAAI solution is placed in the connection circuit between the MPC_PE_ points (connection points for the PE conductors of the power supply cables) and the PG points of the two audio devices. The PG point, located in the rectifier section of each audio device, represents the point of lowest impedance within the device. The corresponding connections, rectifier blocks and other basic circuits that are important for understanding the operation of the source and receiver audio devices and their technical interaction are detailed in the block diagram of the comprehensive audio system model in Figure 1.

The AISAAI utilizes the principle of interpolation of passive, linear and non-linear elements to modify the impedance of this connection to reduce the influence of parasitic currents and voltages caused by audio interference.

**Figure 3:**
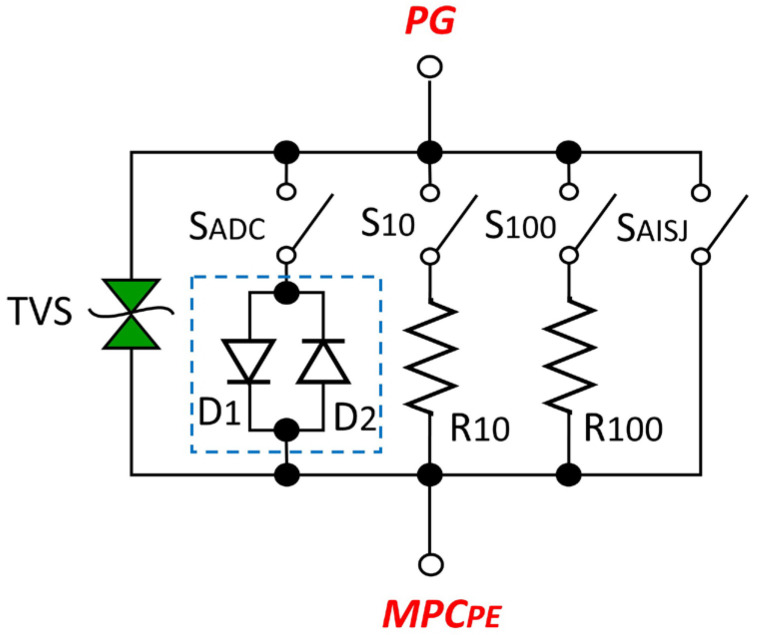
Laboratory setup for analyzing the spectral influence of interpolated suppressors.

In order to experimentally investigate the influence of individual components within the AISAAI suppressor (resistors with different values, diodes) and to determine the optimum configuration for effective audio suppression, a special laboratory setup was developed, which is shown in Figure 3.

**Figure 4:**
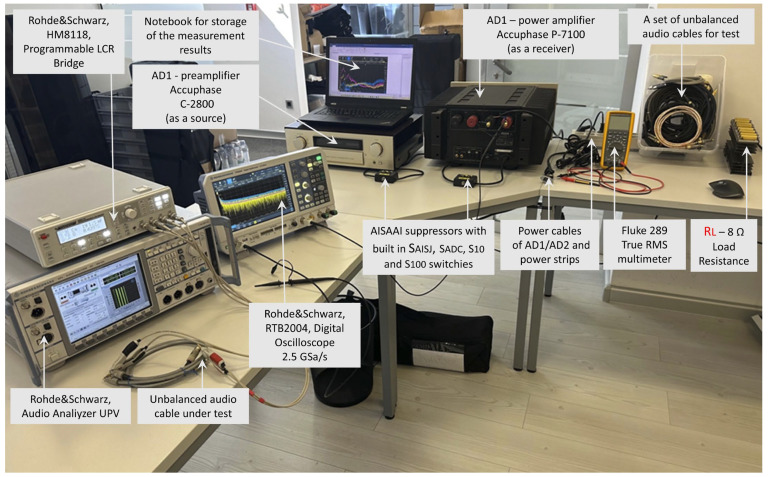
Experimental setup for measuring the influence of the AISAAI suppressor and for cable comparison.

The proposed circuit solution includes the following functional elements:

- S_AISJ_—switch that shorts the AISAAI as needed,
- R_10_—10 Ω resistor with series-connected switch S_10_,
- R_100_—100 Ω resistor with series-connected switch S_100_,
- ADC—antiparallel connection of two diodes, D_1_ and D_2_, indicated by a blue dashed square in Figure 4,
- S_ADC_—switch for engaging the antiparallel connection of diodes D_1_ and D_2_,
- TVS—Transient Voltage Suppressor, providing additional assurance of a low touch voltage level on the audio device chassis (a redundant safety component to the antiparallel diode connection D_1_ and D_2_).

This modular design of the laboratory set-up with the option of selectively switching on individual elements via switches (S_AISJ_, S_10_, S_100_, S_ADC_) made it possible to precisely analyze the contribution of the individual components to interference suppression. By measuring the signal spectrum at an output load RL of 8 Ω, which is a typical load for audio power amplifiers, under different active interference suppression configurations, it was possible to evaluate the effectiveness of the individual elements independently of each other. The measurement results obtained at the output load RL are of fundamental importance for the influence of audio interference on the effect perceived by the listener.

The influence of the power supply configuration was also investigated during the preliminary measurements, using the standard L/N/PE conductor arrangement at the PC_1_ and PC_2_ terminals as well as configurations with reversed L and N conductors [11,22]. This allowed a comparative analysis between standard and non-standard connections. The results show that the position of the phase conductors themselves has only a limited influence, while the topology and the quality of the PE conductors play a decisive role in the level of induced EMI. The detailed results of these investigations are described below.

### 4.1. Experimental Setup and Methodology for the Spectral Analysis of Audio Interference

For the experimental analysis of audio interference, specially structured measurements were carried out to quantify the influence of the individual interpolation elements within the AISAAI suppressor. In addition, a spectral analysis was performed to determine how different power supply configurations and ground connections affect the interference level at the system output.

The measurement methodology was divided into two main stages. In the first stage, preliminary measurements were conducted to characterize the interference sources by analyzing the spectral content of the voltage between the PE points of the power cables, as detailed in Section 4.2.

In the second stage, the main experimental validation measured the effect of this interference at the output of the complete audio system. Measurements were taken across an 8 Ω resistive load (RL) connected to the receiver. A consistent high-fidelity audio system was used for all tests to ensure repeatability. The signal source (AD_1_) was an Accuphase C-2800 preamplifier (Accuphase Laboratory, Inc., Yokohama, Japan), and the receiver (AD_2_) was an Accuphase P-7100 power amplifier (Accuphase Laboratory, Inc., Yokohama, Japan). By utilizing the same source and receiver for all comparative measurements (e.g., comparing different cables or testing with the AISAAI suppressor active vs. inactive), the influence of their respective interface characteristics was held constant. This controlled setup ensures that measured differences in audio interference can be directly attributed to the component under investigation.

The measurements were performed with the following types of measurement instruments:

Measurements of the basic parameters of the effective voltage and current values between the GND connection points of different wall outlets and different topologies of the household power distribution network were performed using a Fluke 289 True RMS multimeter (Fluke Corporation, Everett, WA, USA).

Measurements of parameters of potentially induced parasitic signals in audio cables as a result of external EMI and the potential detection of non-linear distortions possibly generated by the audio cables themselves were performed with a Rohde & Schwarz Audio Analyzer UPV, DC—250 kHz (Rohde & Schwarz GmbH & Co. KG, Munich, Germany).

The measurement of the basic RLC parameters of audio cables was performed with a Rohde & Schwarz HM8118 programmable LCR bridge (Rohde & Schwarz GmbH & Co. KG, Munich, Germany).

The measurement of the amplitude and phase characteristics of audio cables to determine the influence of the parasitic capacitances of the audio cables and the corresponding impedances of the source and receiver in the audio system was performed with a Rohde & Schwarz RTB2004 digital oscilloscope, 2.5 GSa/s (Rohde & Schwarz GmbH & Co. KG, Munich, Germany).

Using the same type of instrument, the Rohde & Schwarz RTB2004 digital oscilloscope, 2.5 GSa/s, the spectra of the signals of interest were recorded with a specific vertical sensitivity and resolution, as well as frequency range (typically up to 20 kHz or 100 kHz, in selected measurements up to 6 MHz). The setting parameters for each recorded spectrum are visible on each individual display. In addition, for each recorded spectrum, the measurement process was repeated several times and averaged, and the labeling of the graphical representation indicates whether it is a curve of average values or, for example, of signal peak values. For each spectrum shown, the author also saved the corresponding measured numerical values, which have been omitted in this text due to their length.

When analyzing the spectrum, the influence of different power supply configurations, such as the connection of devices with mains cables to the same or different sockets or socket strips, and variations in the termination of the ground loop were considered. The contribution of the individual components of the AISAAI suppressor—resistors with values of 1 Ω, 10 Ω and 100 Ω as well as anti-parallel diodes—to the level of induced interference was evaluated.

The measurements focused on identifying the spectral components of the noise induced in the audio signal, focusing on the occurrence of hum at 50 Hz and its harmonics, high-frequency EMI components and their noise products. The system was powered via standard power cords (labeled PC_1_ and PC_2_) and industry standard IEC320 C13 plugs were used for the audio devices. Throughout the process, the standard arrangement of phase (L), neutral (N) and PE conductors was consistently maintained across the entire power path—from the wall sockets to the power strips to the device connections.

In addition, the sequence of the phase conductors L and N was also measured at the connections of the AD_1_ and AD_2_ audio devices. Contrary to accepted professional audiophile recommendations, it was found that swapping the L and N conductors, either on the PC_1_ or PC_2_ cable, did not cause any significant change in the EMI values in the analyzed measurement configuration.

The spectral analysis was primarily performed by measuring the audio interference across the load resistor R_L_, which represents the standard load impedance of the AD_2_ device’s analog output, i.e., the standard loudspeaker load impedance of the power amplifier. According to the block diagram in Figure 1, the level of audio interference at the system output and its dependence on the interpolation elements used and the power supply configuration could thus be directly observed.

The diagram shows the connection of the main components of the audio system under test: the signal source (AD_1_) and the receiver (AD_2_) via an audio connection cable, the output load (R_L_) via which the resulting signal is measured, and the key element for interference suppression—the AISAAI suppressor—which is inserted in the connection circuit between the MPC_PE_ points (connection points for the PE conductors of the power supply cables, i.e., their respective mains plugs to the power supply cables and their respective mains plugs on the socket) and the rectifier PG points of the audio devices AD_1_ and AD_2_.

Also shown schematically are the separate power connections (PC_1_, PC_2_), the input point for the excitation signal (at AD_1_) and the output measuring point (at RL), which form the basis for all measurements carried out.

The physical realization of the ESAAI laboratory setup is shown in Figure 4. The photo shows the implementation of the circuit with accessible switches for the configuration of different interpolation elements, which allowed a detailed comparison of their influence on the overall EMI.

### 4.2. Measuring the Influence of Separate Power Cable Connections for AD_1_ and AD_2_

To analyze the influence of separate power cable connections on the occurrence of audio interference, spectral measurements were carried out between the points AC_PEPC1_ and ACP_EPC2_, which correspond to the PE points of the power cables PC_1_ and PC_2_. These points represent the reference potential of the AD_1_ and AD_2_ audio devices, and the measurement included the spectrum of EMI induced by the galvanic connection and the ground loop. For all measurements performed, the connection of the power cables to the same or different sockets was switched using the SPS switch, as shown in the block diagram in Figure 1. If power strips were required, these were connected in series with the respective power cables.

The measurements were carried out in a closed electrical configuration with the SAC_1_, SAC_2_ and S_PET_ switches turned off to eliminate internal sources of interference and ensure clear comparison conditions. The measurements covered a frequency range up to 6 MHz, focusing on the differences in signal amplitudes in the frequency range up to 2 MHz. The block diagram of the system shown in Figure 1 illustrates the arrangement of the PC_1_ and PC_2_ power cables and the way in which the PE points were connected.

**Figure 5:**
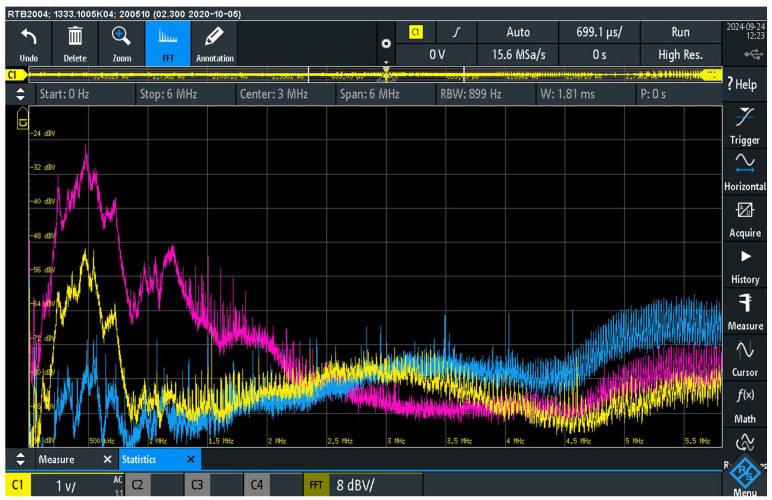
Spectral measurements of audio interference: pink—different power strips on different sockets, yellow—different power strips on the same socket, blue—the same power strip on one socket.

Figure 5 shows the results of measurements carried out in three different configurations of the connection of the PC_1_ and PC_2_ power cables to the power supply. Power strips are regularly encountered in audio systems as several devices need to be connected to the mains voltage, so their use was considered in this analysis. The first configuration (pink curve) corresponds to the connection of the mains cables to different power strips, each connected to a different socket, and shows the average value of the audio interference spectrum, i.e., hum, noise and interference caused by EMI mechanisms between points AC_PEPC1_ and AC_PEPC2_. The second configuration (yellow curve) corresponds to connecting the power cables to different power strips, both connected to the same wall socket, and also shows the average voltage spectrum. The third configuration (blue curve) corresponds to the connection of both power cables to the same power strip connected to a single wall socket and shows the peak voltage spectrum of the audio interference. The peak voltage representation was chosen in this case because this configuration showed the lowest audio interference up to a frequency of 2 MHz and to make the graphical representation comparable with the other two configurations within a clearly visible vertical scale. For orientation, the difference in the absolute values between the peak and average voltage curves in this diagram is about 10 dB on average.

These measurements confirm the occurrence of audio interference between the ACPEPC_1_ and AC_PEPC2_ points, i.e., the PE conductors of the PC_1_ and PC_2_ power cables, which in a real audio system environment are assigned to the MPC_PE_ points of the power plugs (MPC) on the housing of the AD_1_ and AD_2_ audio devices. The observed EMI shows significant amplitude differences up to a frequency of 2 MHz, while the signals above this range show almost uniform levels. Consequently, the observed EMI during operation of the audio system implies audio interference as a result of the galvanic connection of the PE conductors to the signal ground within the audio device, together with the simultaneous occurrence of audio interference through the negative conductor of the unbalanced audio cable. As a result, the useful signal is superimposed by the audio interference, which leads to its deterioration.

The measurement results clearly indicate a significant influence of the electrical topology of the power cable connections on the degree of audio interference [8]. The measurements were carried out with standard power cords that are usually supplied with the audio equipment itself. More sophisticated designs of audio power cords and their different technical designs usually change the level of induced EMI in the protective conductor. This consideration is the subject of a separate study. It is particularly important to consider the type of connection of PE points (common or separate) in order to minimize the negative effects of audio interference on the quality of audio transmission.

### 4.3. Measuring the Influence of Ground Loop Termination

In order to analyze the influence of different ground loop termination configurations on the occurrence of EMI in the audio system, measurements were performed to investigate the behavior of the system in the case of terminated and non-terminated ground loops.

For the measurement, two earthing points labeled AC_PEPC1_ and AC_PEPC2_ were used, which represent the PE points of the power cables PC_1_ and PC_2_. The PC_1_ and PC_2_ power cables were connected directly to different wall sockets, with no power strips present, and all switches in the laboratory system, including SAC_1_ and SAC_2_, were switched off. The measurement covered a frequency range of up to 3 kHz and focused on the fundamental frequency of the supply voltage and its higher harmonics, with an emphasis on analyzing the effects of EMI when the ground loop was broken.

**Figure 6:**
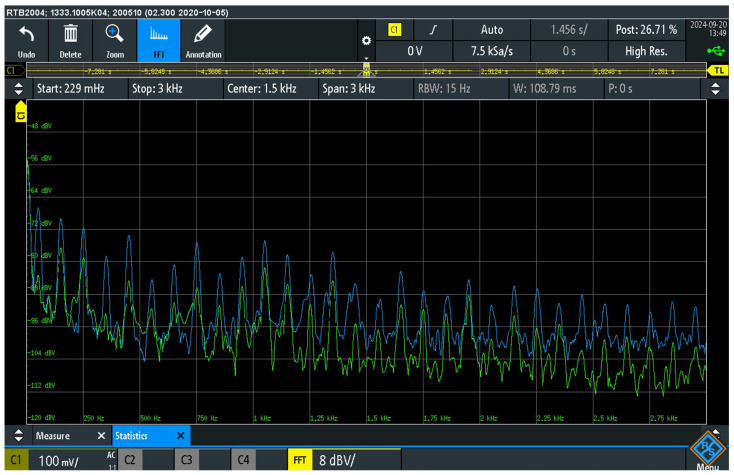
Spectral measurements of the influence of ground loop termination on the EMI: Blue—non-terminated loop, green—terminated loop.

Figure 6 shows the spectral measurements of audio interference between the AC_PEPC1_ and AC_PEPC2_ points for a non-terminated and a terminated ground loop.

In the disconnected ground loop configuration, power cables PC_1_ and PC_2_ were connected to different sockets. In this situation, the ground loop was not completed (S_PET_ switch off), resulting in higher interference amplitudes, especially at low frequencies.

To complete the ground loop, the power cables of PC_1_ and PC_2_ were also connected to different sockets, and the S_PET_ switch was turned on so that a 1 Ω resistor was connected between points AC_PEPC1_ and AC_PEPC2_. This resistance simulates the approximate resistance found in a real audio system and in the grounding system configuration, taking into account all elements: the protective grounding wires inside the audio equipment, the contact resistances of the MPCPE_1_ power supply connectors on the chassis of the AD_1_ and AD_2_ audio equipment, the resistance of the internal protective grounding wires, the contact resistances of the negative conductor terminals of the audio cable and the resistance of the negative conductor of the audio cable. Compared to the undermined loop, a reduction in the level of the spectral components of the audio interference was observed in this setup.

The measurements showed that spectral harmonics of audio interference occur between points AC_PEPC1_ and AC_PEPC2_ due to the galvanic connection between the cables and the signal grounds of the audio devices, including the negative conductor of the audio cable. The interference occurred not only in a broader frequency band, as shown in Figure 6, but mainly in the frequency range up to 3 kHz, indicating that the galvanic connection between the PE points allows a significant injection of audio interference into the system via the grounding.

The graph clearly shows that the terminated loop with the 1 Ω resistor (green curve) reduces the amplitude differences compared to the non-terminated loop (blue curve), which is reflected in a reduction in the peak voltages of the ground loops by around −10 dB. The measurement with the terminated ground loop was performed to demonstrate the coupling of audio interference under real operating conditions of an audio system, i.e., with a realistic load impedance of the ground loop, focusing on the detection of hum signals, especially multiples of the mains frequency of 50 Hz.

### 4.4. Measurement of the Influence of the AISAAI Suppressor with R = 10 Ω on the Unbranded NBAC Cable

The measurement of the influence of the AISAAI suppressor on the unbranded NBAC cable was performed to evaluate the effectiveness of the application of a suppressor in audio systems, particularly in the context of audio interference reduction. The system configuration included the measurement of audio interference without and with the influence of the suppressor with R = 10 Ω. The measurements were performed at the output of the AD_2_ audio device according to the block diagram shown in Figure 1.

The measurement was carried out in the frequency range up to 20 kHz. The AD_1_ and AD_2_ audio devices were connected via an unbranded audio cable, and the PC_1_ and PC_2_ power cords were connected to different power strips, each with separate power strips, creating a potential difference in the protective grounding system. The SAC_1_, SAC_2_, SAD_1_ and SAD_2_ switches were switched on, while the S_PET_ switch was switched off. The input of the AD_1_ device was terminated with a resistor R_IN1_ of 60 Ω, which is typical of the output impedance of a previous audio signal source, with the S_IN1_ switch on. The volume potentiometer on the AD_1_ device was set to its minimum to eliminate the influence of the internal gain on the measurement results.

**Figure 7:**
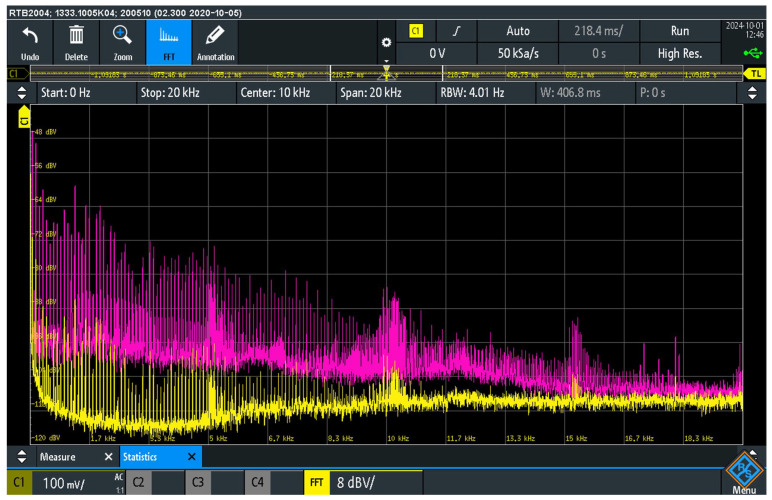
Spectral measurements of unbranded cables with suppressor R = 10 Ω: pink—pre-suppression (off), yellow—post-suppression (on).

Figure 7 shows the pre- and post-suppression spectrum of audio interference at the load resistor R_L_ at the output of the audio device AD_2_. The measurement was carried out in two suppressor configurations. For the deactivated suppressor, the S_AISJ_ switch in the suppressor was switched on (pink curve), short-circuiting and deactivating the suppressor. In the second configuration, only switch S_10_ in the suppressor was switched on. In this configuration, the suppressor was active (yellow curve), whereby the 10 Ω resistor was activated.

The measurement results show a significant reduction in the level of audio interference in the presence of the interference suppressor with a 10 Ω resistor at the load R_L_ of the output of the AD_2_ audio device. In the frequency range up to 5 kHz, the signal level was reduced by −30 dB. In the 5–10 kHz range, the reduction was about −20 dB, while in the 10–20 kHz range it was about −15 dB. These results confirm that the use of the suppressor significantly reduces the induced EMI. The results indicate a significant contribution of the suppressor to the reduction of audio interference, which is crucial for maintaining high signal transmission quality in audio systems.

### 4.5. Measurement of the Influence of the AISAAI Suppressor with R = 100 Ω and Diodes in Anti-Parallel Circuit on the Unbranded NBAC Cable

The measurement was carried out in accordance with the measurement setup described in the previous section.

**Figure 8:**
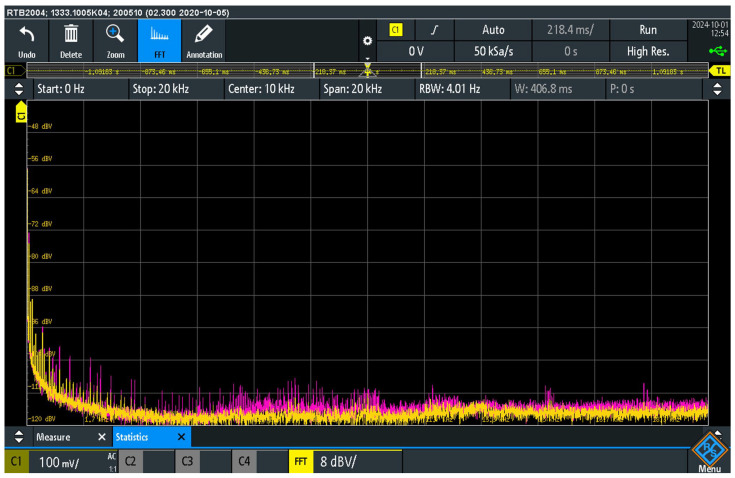
Spectral measurements of the influence of the AISAAI suppressor with R = 100 Ω and diodes in an anti-parallel circuit on the unbranded cable: pink—suppressor with R = 100 Ω; yellow: diodes in an anti-parallel circuit.

Figure 8 shows the spectrum of the audio interference at load resistor R_L_. The measurement was carried out in two suppressor configurations. In the first configuration with an interpolated 100 Ω resistor (pink curve), switches S_AISJ_, S_10_ and S_ADC_ were switched off, while switch S_100_ was switched on. In this configuration, the suppressor was activated with an interpolated 100 Ω resistor. Compared to the previous configuration R = 10 Ω, a further reduction in the level of audio interference was observed. In the frequency range up to 2 kHz, the interference level was reduced by −20 dB, up to 10 kHz the reduction was −15 dB and up to 20 kHz −10 dB.

In the second configuration with activated antiparallel diode switching, the S_ADC_ switch was switched on, thus activating the antiparallel switching of two diodes (yellow curve) in the interference suppressor, while all other switches S_AISJ_, S_10_ and S_100_ were switched off. The measurement results of this configuration show the occurrence of audio interference with non-linear elements in the return path. Compared to the configuration with the interpolated 100 Ω resistor, a further reduction in the level of audio interference can be seen. In the frequency range up to 4 kHz, the interference level was reduced by −10 dB, up to 10 kHz the reduction was −6 dB and up to 20 kHz −4 dB.

The quantitative results of the interference suppression for each preliminary configuration, as discussed in Section 4.4 and Section 4.5, are summarized in Table 3 for a direct comparison.

The measurement results confirm the significant presence of parasitic audio interference signals at the load resistor R_L_, whereby the interference level is strongly dependent on the elements used in the interference suppression circuit. The comparison between the use of a 100 Ω resistor and the anti-parallel diode circuit shows that the use of non-linear elements further reduces the level of audio interference. The introduction of diodes allows additional limitation of the audio interference caused by galvanic connections in the system.

These results suggest that the interpolation of non-linear elements, such as the use of diodes in an anti-parallel circuit, may have advantages over simple resistor methods in reducing the level of audio interference, particularly in sensitive audio systems. However, further considerations show that a specific combination of linear and non-linear electronic elements is required for the practical application and proper functioning of touch voltage protection on the chassis of audio systems.

## 5. Configuration of the AISAAI Suppressor Based on Audio Interference Analysis

A comparative analysis of the signals shown in Figure 7 and Figure 8 shows the injection of audio interference into the signal ground of the source audio device AD_1_ and the detection of this interference at the load resistor R_L_ of the audio device AD_2_. It has been determined that this injection occurs through a galvanic connection mechanism. As a result, audio interference occurs in the negative conductor of the audio cable. The voltage drops caused by these currents is superimposed on the input signal of the AD_2_ audio device. After amplification in the AD_2_ device, this superimposed audio interference manifests itself at the output load R_L_.

**Figure 9:**
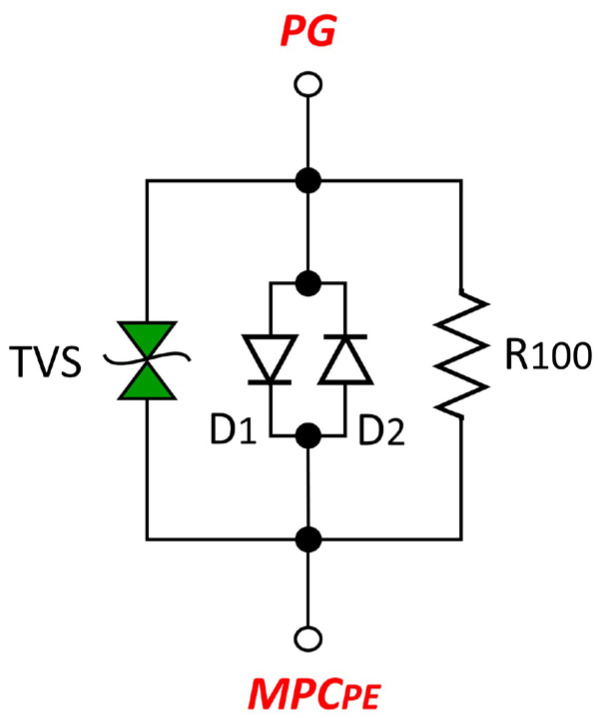
AISAAI Audio Interference Suppressor.

These measurements clearly show that the level of the audio interference detected at the output is highly dependent on the impedance of the interference suppressor connected between the chassis of the AD_1_ and AD_2_ devices and the PE. Although various solutions and circuit topologies for grounding the enclosure and suppressing audio interference are known and used in practice, the comparative measurements carried out as part of this research identified a parallel combination of a resistor R_S_ = 100 Ω and anti-parallel diodes (D_1_, D_2_) as the configuration that provides the most effective suppression of audio interference observed in this case (Figure 9).

Therefore, based on the experimental results, this parallel configuration was chosen for all further measurements in this paper. This configuration also fulfills the practical need to divert potential static voltages from the device chassis to the protective conductor [16,21], a function that is also fulfilled by other grounding concepts.

The final optimized AISAAI circuit, shown in Figure 9, consists of the following components:

- R_100_: A 100 Ω resistor (e.g., a standard 1 W metal film type).
- D_1_, D_2_: Two standard silicon rectifier diodes (e.g., UF600M) connected in an anti-parallel configuration to ensure a low-impedance path for fault currents.
- TVS: A bidirectional Transient Voltage Suppressor diode with a breakdown voltage selected to be safely above the peak mains voltage, providing additional fault protection.

The function of the R_100_ = 100 Ω resistor in the suppressor has been identified as twofold. First, it serves to suppress audio interference in the audio equipment signal grounds, which primarily affects the negative conductor of the unbalanced audio cable. Secondly, it provides a continuous path for the dissipation of induced static charge from the housing of the audio equipment via a galvanic connection from point EP to PG and then via resistor R_100_ to point MPC_PE_ of the mains plugs on the housing of both audio devices, i.e., to PE. The occurrence of static charge on the chassis of the audio equipment is a result of EMI from the mains cables inside the audio equipment, stray fields from power transformers, pulse currents for charging electrolytic capacitors in the rectifier, external parasitic electromagnetic fields, etc.

The main task of the anti-parallel diodes (D1, D2) in the interference suppressor is to dissipate high fault currents: In the event of an internal fault where the outer conductor (L) of the power supply system comes into galvanic contact with the device chassis, the diodes create a low-impedance path for the resulting high fault current to the protective conductor [4]. In view of the connection of the PE and N conductors in the distribution board, this creates a short circuit between L and N, which causes the protective fuses to trip quickly. In addition, the diodes also serve to limit overvoltages by discharging parasitic voltages induced on the chassis to the PE conductor if these voltages exceed the forward threshold of the diodes.

The diode element for suppressing voltage spikes (TVS), which is connected in parallel with the resistor and the anti-parallel diode circuit, has a redundant safety function within the AISAAI circuit. In the event of an overvoltage that exceeds a certain breakdown voltage, the TVS element can conduct an extremely high fault current or short-circuit current. In the event of a malfunction or burnout of the TVS element due to high heat dissipation, its technical design allows it to act as a short-circuiter and galvanically connect the chassis of the audio device directly to the PE. In this way, the AISAAI circuit fully ensures the function of suppressing audio interference in the signal grounds of audio devices and at the same time provides the safety function of conducting the short-circuit current in the event of a fault within the audio device.

### 5.1. Experimental Validation of the Efficiency of the AISAAI Suppressor

After a preliminary analysis of the influence of individual suppression components on audio interference, in which measurements were performed without an external excitation signal and with minimal gain to isolate the active contribution of the components, an experimental validation of the selected optimal AISAAI suppression configuration was performed under conditions closer to actual operation. For this reason, a defined external audio signal of 1 kHz was used for all measurements described below. In this way, the effectiveness of the suppressor can be evaluated not only in terms of reducing the background level of audio interference, but also in relation to the presence of a wanted signal, including the potential impact on increased interaction between the audio interference and the signal, such as intermodulation distortion in audio equipment.

To experimentally confirm the effectiveness of the previously optimized AISAAI suppressor, measurements were performed at the output of the audio system. The system consisted of the audio device AD_1_ (signal source/preamplifier) connected to the audio device AD_2_ (signal receiver/power amplifier), with the output signal measured via a load resistor R_L_ = 8 Ω as in previous measurements.

The test signal was a sinusoidal signal with a frequency of 1 kHz and an amplitude of 100 mV_pp_ generated by a signal generator with a low output impedance of 50 Ω (SIN_1_ off).

The gain of the AD_1_ device was adjusted so that the level of the fundamental (1 kHz) at the load R_L_ was −20 dBV. A key element of the test setup was the type of power supply: The power cords of the AD_1_ (PC_1_) and AD_2_ (PC_2_) devices were connected to different wall sockets with the standard arrangement of L and N conductors to simulate a realistic situation that tends to produce a slightly larger ground potential difference than, for example, a topology where the power cords are connected to the same wall socket.

The AISAAI suppressor was connected between PG and MPC_PE_ points, as shown in Figure 1. Its activation was controlled by the S_AISJ_ switch: In the closed position, the switch short-circuits the suppressor, while in the open position, it is included in the connection circuit between PG and MPCPE. Other relevant system switches (SAC_1_, SAC_2_, SAD_1_, SAD_2_) were switched on and the S_PET_ switch was switched off. The measurements were performed with two types of unbalanced audio cables: a generic (NB—Non-Branded) NBAC and a reference cable (Acc—Accuphase) ASRC.

#### 5.1.1. Measuring the Influence of the AISAAI Suppressor on the Unbranded Cable

The measurement of the influence of the AISAAI suppressor on the unbranded NBAC cable was performed to evaluate the effectiveness of this particular suppressor in audio systems, especially in the context of audio interference reduction. The system configuration included the measurement of audio interference without the influence of the suppressor and with the influence of the active AISAAI suppressor.

The measurements were carried out at the output of the AD_2_ audio device.

The measurements were performed in the frequency range up to 20 kHz using the non-branded NBAC audio cable to connect the AD_1_ and AD_2_ devices. The most important condition for testing the effectiveness of the suppressor under real conditions, namely, the simulation of an earth potential difference, was achieved by supplying the AD_1_ (via PC_1_) and AD_2_ (via PC_2_) devices with power via separate socket strips. The excitation signal was a sine wave voltage of 1 kHz (100 mV_pp_, R_g_ = 50 Ω) from a signal generator applied to the input of AD_1_ (SIN_1_ off), and the signal level at the output load R_L_ was set to −20 dBV using the gain control on AD_1_. The switches SAC_1_, SAC_2_, SAD_1_ and SAD_2_ were switched on and S_PET_ was switched off.

**Figure 10:**
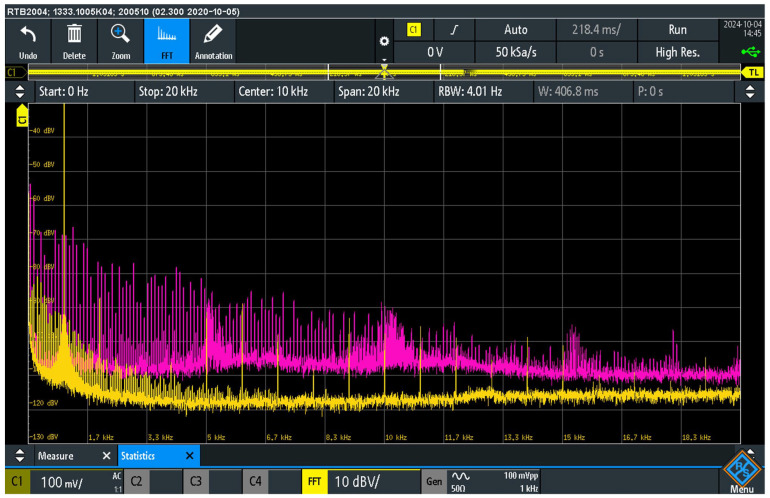
Spectral measurements of the influence of the AISAAI suppressor on the unbranded cable: pink—pre-suppression (off), yellow—post-suppression (on).

Figure 10 shows the pre- and post-suppression signal spectrum at the load resistor R_L_ at the output of the audio device AD2. The measurement was carried out in two configurations in relation to the activity of the interference suppressor. In the first configuration (pink curve), the S_AISJ_ switch in the suppressor was switched on, short-circuiting and deactivating the suppressor.

In the second configuration, the S_AISJ_ switch in the suppressor was switched off. In this configuration, the suppressor was active (yellow curve), with a parallel combination of a 100 Ω resistor and anti-parallel diodes.

The measurement results show that the level of audio interference decreases significantly in the presence of the active AISAAI suppressor. In the frequency range up to 5 kHz, the noise level was reduced by around 30 dB. In the range from 5 to 10 kHz, the reduction was about 25 dB, while in the range from 10 to 20 kHz it was about 15 dB.

#### 5.1.2. Measuring the Influence of the AISAAI Suppressor on the Accuphase ASRC Cable

The measurement of the influence of the AISAAI suppressor, this time using the Accuphase Super Refined reference audio cable, was carried out to further assess the effectiveness of the suppressor and the influence of cable quality on the reduction of audio interference. As in the previous case, the system configuration included a comparison of audio interference measurements with and without the influence of the AISAAI suppressor. The measurements were performed at the output of the AD2 audio device.

The AD_1_ and AD_2_ audio devices were connected with the ASRC cable under identical conditions to the previous measurement with the NBAC cable.

**Figure 11:**
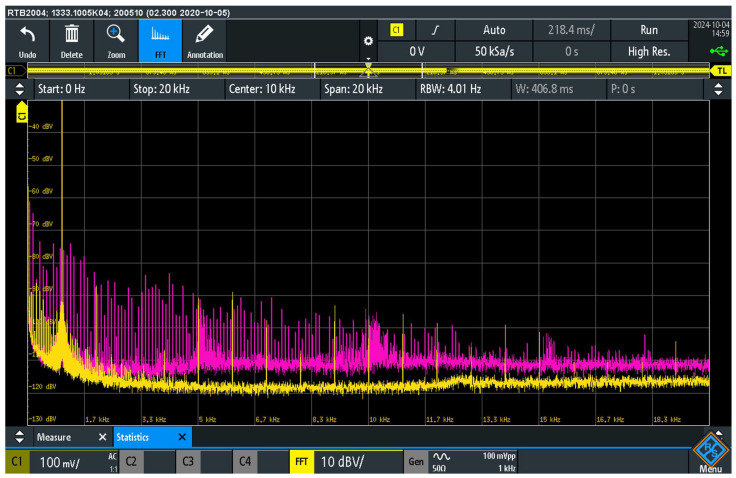
Spectral measurements of the influence of the AISAAI suppressor on the Accuphase cable: pink—pre-suppression (off), yellow—post-suppression (on).

Figure 11 shows the pre- and post-suppression signal spectrum at the load resistor R_L_ for two configurations in relation to the activity of the AISAAI suppressor, identical to the previous test of the NBAC cable.

For clarity, the key performance metrics of the final AISAAI suppressor on both the unbranded (NBAC) and Accuphase (ASRC) cables are consolidated and presented in Table 4.

The measurement results show that the level of audio interference decreases significantly in the presence of the active AISAAI suppressor, even when using the high-quality Accuphase cable. With the suppressor inactive (pink curve), the higher harmonics of the wanted signal up to 5 kHz are still completely masked by the audio interference, although some harmonics in the higher frequency band can be partially detected.

When the suppressor is activated (yellow curve), the level of the interfering signals is reduced by around 25 dB in the range up to 5 kHz, by 20 dB in the range from 5 kHz to 10 kHz and by 10 dB in the range from 10 kHz to 20 kHz.

The noise floor curve is also significantly lower when the suppressor is activated. Comparing these results with those of the NBAC cable, as shown in Figure 10, it is noticeable that the absolute noise levels are slightly lower, and, therefore, the overall result is slightly better when ASRC is used, although the effectiveness of the suppressor itself (expressed in dB reduction) is slightly lower in some parts of the spectrum. This confirms that the AISAAI suppressor provides a significant improvement regardless of the cable used, but also that a higher-quality cable can itself contribute to a lower initial noise level.

#### 5.1.3. Comparison of the Influence of the Quality of the Audio Cable on the Audio Interference Without Active Suppressor

This measurement was carried out with the aim of directly comparing the influence of two different audio cables (unbranded and Accuphase) on the level of audio interference, but without the effect of the AISAAI suppressor. The aim was to evaluate the inherent differences between the cables in terms of transmission or generation of interference in the given system configuration. The measurements were performed at the output of the AD_2_ audio device (at a load resistance R_L_ = 8 Ω) according to the block diagram in Figure 1.

The measurements were carried out in the frequency range up to 20 kHz. The AD_1_ and AD_2_ audio devices were first connected to the NBAC and then to the ASRC audio cable. The main condition for the simulation of ground potential differences (supplying the AD_1_ (PC_1_) and AD_2_ (PC_2_) devices via separate power strips) remained the same as in previous measurements. An excitation signal of 1 kHz (100 mV_pp_, R_g_ = 50 Ω) from a signal generator was applied to the input of device AD_1_ (SIN1 off), and the gain of AD_1_ was set to an output level of −20 dBV of the fundamental at load RL. The state of the other system switches was as follows: SAC_1_, SAC_2_, SAD_1_, SAD_2_ on; S_PET_ off. It should be noted that the S_AISJ_ switch was switched on for both measurements with NBAC and ASRC.

**Figure 12:**
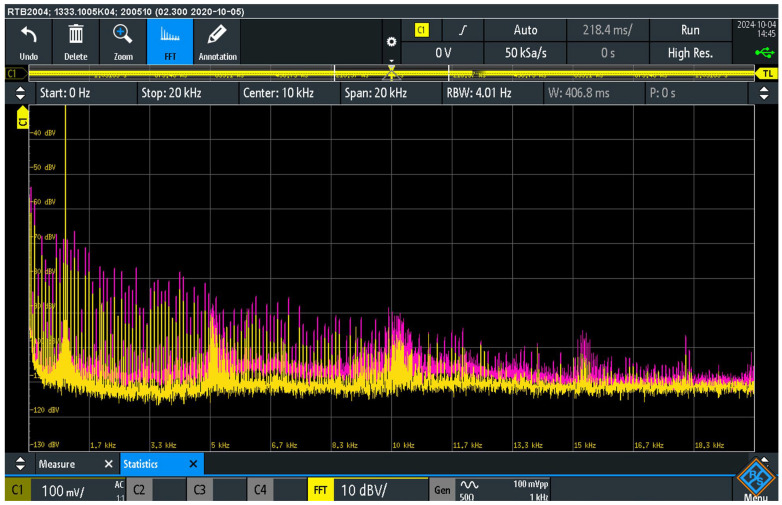
Comparison of the audio interference spectra when using different audio cables (AISAAI suppressor inactive): pink—NB cable; yellow—Accuphase cable.

Figure 12 shows comparative signal spectra at load resistor RL obtained to compare the influence of the audio cable used with an inactive AISAAI suppressor. Two spectra are shown: one corresponds to the configuration with NBAC (pink curve), the other to the configuration with the ASRC audio cable (yellow curve).

The measurement results show the influence of the audio cable type on the noise and interference level when the interference suppressor is not active. In both cases, the fundamental signal of 1 kHz is visible as well as a broad spectrum of EMI signals that mask the higher harmonics of the wanted signal.

However, a comparison of the curves clearly shows the differences depending on the cable used. The use of the Accuphase Super Refined cable results in a lower level of audio interference compared to the unbranded cable. In quantitative terms, the comparable reduction in the noise level is around 10 dB up to a frequency of 10 kHz and around 5 dB in the range from 10 to 20 kHz. The noise floor is also 5–7 dB lower when using the Accuphase cable in the range up to about 12 kHz, while above this frequency the noise floor curves converge. These results show that the ASRC audio cable offers better protection against audio interference, regardless of the effect of the AISAAI suppressor.

## 6. Discussion

The results presented in this paper provide a quantitative insight into the problem of EMI in unbalanced audio systems and demonstrate the effectiveness of the proposed AISAAI suppressor. The proposed AISAAI suppressor is not the only available method for reducing audio interference, and it is important to compare it with existing approaches. In professional environments, direct injection (DI) boxes and audio isolation transformers are widely used to provide galvanic isolation and thereby eliminate ground loops. While effective, they can introduce drawbacks such as frequency response limitations, phase distortion, and additional cost, which may be undesirable in high-fidelity audio applications. DC blockers, placed in the mains power line, are another common solution and can reduce mechanical hum caused by transformer core saturation. However, they do not address the low-frequency ground loop hum that directly contaminates the audio signal. Finally, filtering methods such as common-mode chokes or capacitive filters can attenuate higher-frequency interference but are less effective against the 50/60 Hz hum and its harmonics that dominate in unbalanced systems [1]. Compared to these approaches, the AISAAI suppressor provides a simple, passive, low-cost, and safe hardware solution that directly mitigates ground loop interference in the analog audio interface without degrading audio fidelity.

Objective characterization confirmed significant variations in the RLC parameters of commercially available audio cables, with the impedance of the return conductor differing by orders of magnitude, primarily due to variations in resistance and inductance Analysis of interference sources clearly identified the common-mode voltage between the PE terminals of the devices, due to the topology of the mains power supply, as a major driving mechanism for interference. Experiments with the termination of the ground loop also confirmed that this loop, which includes the return conductor of the cable, is the primary path for interference injection.

The key finding is that the proposed AISAAI circuit in its optimized configuration (a 100 Ω resistor in parallel with anti-parallel diodes) inserted into the connection between chassis/signal ground and PE consistently reduces hum and noise levels at the system output by a substantial 15–30 dB within the audio frequency band. This effectiveness has been proven with connection cables that have very different impedance characteristics (e.g., ASRC and NBAC).

The suppression mechanism of the AISAAI can be interpreted by specifically changing the impedance of the ground loop. By inserting a relatively high resistor (100 Ω) for low-amplitude interference signals (typical for hum and noise), the AISAAI limits the common-mode current flow induced by PE potential differences. This reduced current in turn results in a lower interference voltage drop across the return conductor impedance of the audio cable, which minimizes the superimposition of interference signals on the useful signal. Analysis of the individual AISAAI components shown in Figure 8 and Figure 9 suggests that while suppression is provided by the reactance resistors alone, the addition of anti-parallel diodes offers a further improvement [20,21], probably by intercepting voltage spikes of the interference, in addition to the crucial safety function of providing a low impedance path for fault currents. The observed decrease in the interference suppression effect at higher frequencies requires further investigation but can be attributed to parasitic capacitances and the frequency characteristics of the AISAAI components.

These findings contribute to the ongoing discussion about the influence of cables on sound quality [8,10]. The results clearly show that while cables with lower return path impedance (such as ASRC) inherently have lower noise levels in the presence of ground loops, the critical factor is the interaction of the cable with the overall system, particularly the grounding scheme and common-mode voltages (Figure 12). The AISAAI does not change the cable itself but fixes the problem at the system interface (signal/chassis ground to PE), effectively mitigating the negative effects of the ground loop regardless of the cable used, although the overall result is better with a better quality cable. This suggests that measurements on isolated cables may be inadequate as they do not capture the degradation mechanism prevalent in many real systems. The AAIS concept proved to be a valuable framework for considering these systemic interactions.

Compared to other ground loop mitigation techniques, the AISAAI concept offers significant advantages [4]. In contrast to the unsafe practice of “ground lifting”, AISAAI preserves the integrity of the safety PE connection. Compared to audio isolation transformers, AISAAI is a simpler and potentially more cost-effective passive solution that does not have the potential frequency response limitations or distortion characteristics of transformers, although it does not provide complete galvanic isolation. Compared to active noise suppression circuits, the passive nature of AISAAI and the simplicity of implementation are notable advantages [20,21].

The practical implication of this research is that AISAAI provides a viable method for significantly improving audio quality (reducing hum and noise) in numerous existing or new unbalanced audio systems without the need for expensive cables or complex system modifications. It could be implemented as a stand-alone adapter or integrated into the design of audio equipment.

The robustness of the AISAAI suppressor is expected to be maintained under varied conditions. Because it modifies the impedance of the ground reference rather than the audio signal path, its performance should be independent of varying audio loads (e.g., different loudspeakers). In more complex setups with multiple devices or longer cable runs, the suppressor is expected to provide consistent interference reduction. While longer cables may increase the absolute interference level in an unsuppressed system, the AISAAI’s fixed high impedance continues to dominate the loop, ensuring a consistent suppression ratio. This is supported by our experimental results with cables of widely differing impedances, which already demonstrate their effectiveness in different environments.

However, it is important to recognize the limitations of this study. The investigation focused exclusively on unbalanced connections and a specific configuration of test equipment (AD_1_, AD_2_) in a laboratory environment. Although two cables with different characteristics were used, the validation did not include a broader range of cables with the final AISAAI configuration. Only one AISAAI configuration (100 Ω || diodes) was comprehensively validated. Furthermore, neither correlation with formal subjective listening tests nor a systematic analysis of the behavior at very high frequencies or against standardized EMC immunity tests was performed. The AAIS model used also represents a certain simplification of a real system. The effectiveness of the AISAAI suppressor is maintained under varying load conditions, since it modifies the impedance of the ground reference rather than the audio signal path. Experimental results with cables of widely differing impedances already demonstrate robustness across different environments. In more complex setups with multiple devices or longer cable runs, the suppressor is expected to provide consistent interference reduction, although the absolute interference level prior to suppression may be higher due to increased loop area.

Although this study focused on unbalanced analog interfaces, the principles of the AISAAI suppressor have potential applications in other audio contexts. Balanced interfaces provide strong inherent immunity to ground loop interference due to their differential signaling. However, residual problems can still occur from imperfect component balance or compromised cable shielding [14,17]. Future research could explore adapting the controlled impedance concept of the AISAAI to further mitigate these frequency disturbances. While the benefits are expected to be more subtle than in unbalanced systems, this approach could offer an additional layer of noise suppression in critical applications.

In digital audio interfaces such as AES/EBU, S/PDIF, or USB, interference mechanisms shift toward high-frequency electromagnetic coupling, jitter, and ground potential differences affecting digital signal integrity [1,23,24] The current AISAAI design is not optimized for these high-frequency conditions. However, the underlying principle of managing ground impedance remains relevant and could inform future designs that combine this approach with filtering strategies tailored for high-speed digital transmission.

These limitations pave the way for future research. Logical next steps include investigating the applicability of the AISAAI principle to balanced audio lines, conducting formal subjective listening tests to confirm perceived improvements in audio quality, optimizing AISAAI components for specific applications, testing with a wider variety of audio equipment and in different EMI environments, and evaluating the solution against relevant EMC standards.

## 7. Conclusions

This work successfully addressed the problem of signal degradation due to EMI in unbalanced audio systems, with a focus on ground loops. Through a systematic approach based on the AAIS concept, it was confirmed that the analysis of isolated audio interconnect cables is insufficient, and common-mode voltages originating from the power supply and grounding system were identified as important sources of interference.

As a solution, the passive AISAAI circuit was proposed, analyzed and validated, and it was interpolated into the connection between the MPC_PE_ and the PG points of the audio system. The proposed solution is practical because the same effect is achieved by interpolating the AISAAI circuit between the points of the protective conductor of the ACPEP power supply cable and the connection point of the power connector on the chassis of the MPC_PE_ audio device. This allows easy application by connecting the AISAAI circuit in series with the power cable without the need for physical access to the inside of the audio equipment.

The main findings of the work is the experimental confirmation that the optimal AISAAI configuration (100 Ω in parallel with an anti-parallel diode connection) significantly suppresses EMI and reduces the level of hum, noise and interference in the audio range by 15–30 dB, regardless of the quality of the connection cable used.

Overall, the AISAAI circuit provides a simple, passive and effective solution to mitigate common EMI problems caused by ground loops in unbalanced audio systems. It improves the signal-to-noise ratio by suppressing audio interference while reducing the level of non-linear intermodulation products resulting from their superposition with the audio wanted signal. This leads to an improvement in fidelity while maintaining ground reliability and, together with the AAIS concept, provides a practical contribution to solving signal quality problems in real audio systems. It has been clearly shown that power supply cables for audio equipment are also elements of the AAIS system and that their parameters influence the degradation of the transmitted audio signal. The position of the phase conductors of the power cables has an almost negligible influence, while the physical length, topology and manufacturing technology of the power cables play a decisive role in the level of induced EMI. It is also shown that higher-quality audio cables have a greater resistance to the superposition of audio interference with the wanted signal, while the application of the AISAAI circuit significantly improves the level of audio interference suppression in both cases, using fewer and higher-quality interconnect cables.

In addition to typical two-device connections, the AISAAI suppressor remains effective in more complex audio setups with multiple devices or extended cable runs, ensuring robust mitigation of ground loop interference across a wide range of practical scenarios. These findings underline the AISAAI’s potential as a practical and broadly applicable tool for improving the fidelity of analog audio systems.

The practical implications of this study are twofold. First, manufacturers can integrate the AISAAI suppressor directly into the design of new audio equipment at minimal cost. Second, end-users can apply the AISAAI concept as a stand-alone adapter to existing systems, achieving immediate and measurable improvements in audio fidelity. Beyond these applications, the AISAAI approach may also complement professional audio practices and inspire adaptations for mixed-signal and digital systems where ground noise remains a persistent challenge.

Guidelines for future research include subjective evaluation of the quality of the transmitted audio signal with and without the AISAAI circuit, consideration of the degree of susceptibility to induced EMI through the ground loop in balanced systems, and consideration of the degree of induced EMI as a function of the characteristics and technical design of the power supply cables.

## Figures and Tables

**Table 1:** Comparison of measured resistance, inductance, and capacitance across 12 unbalanced audio cables.

Electrical Parameter	Unit	Minimum Value	Median Value	Maximum Value
Capacitance	pF/m	38.72 (Shunyata Research Aries)	111.35 (AudioQuest Amazon)	1252.53 (Transparent Reference)
Inductance	µH/m	0.27 (Accuphase Super Refined)	3.63 (Transparent MusicLink Ultra)	27.40 (Transparent Reference)
Resistance	mΩ/m	5.54 (Accuphase Super Refined)	16.71 (Transparent Reference)	90.7 (Generic Audio Cable)

**Table 2:** Measured and calculated parameters of the ASRC and NBAC audio cables.

Category	Metric	Unit	100 Hz	1000 Hz	2000 Hz	5000 Hz	10,000 Hz	20,000 Hz
Accuphase Super Refined Cable (L = 1.04 uH/m)	R_ASRC_	Ω/m	0.00554	0.00554	0.00554	0.00554	0.00554	0.00554
	X_L_	Ω/m	0.000653	0.006535	0.013069	0.032673	0.065345	0.130690
	Z_ASRC_	Ω/m	0.0056	0.0086	0.0142	0.0331	0.0656	0.1308
	X_L_/R_ASRC_		0.12	1.18	2.36	5.90	11.80	23.59
	Z_ASRC_/R_ASRC_		1.01	1.55	2.56	5.98	11.84	23.61
Non-Branded Audio Cable (L = 1.65 uH/m)	R_NBAC_	Ω/m	0.0907	0.0907	0.0907	0.0907	0.0907	0.0907
	X_L_	Ω/m	0.001037	0.010367	0.020734	0.051836	0.103672	0.207345
	Z_NBAC_	Ω/m	0.0907	0.0913	0.0930	0.1045	0.1377	0.2263
	X_L_/R_NBAC_		0.01	0.11	0.23	0.57	1.14	2.29
	Z_NBAC_/R_NBAC_		1.00	1.01	1.03	1.15	1.52	2.50
Comparison	Z_ASRC_/Z_NBAC_		0.061	0.094	0.153	0.317	0.476	0.578
	Suppression	dB	−24.22	−20.55	−16.33	−9.97	−6.45	−4.76

**Table 3:** Summary of interference reduction for preliminary suppressor configurations.

Suppressor Configuration	Frequency Range	Approximate Interference Reduction (dB)
10 Ω Resistor (vs. no suppressor)	0–5 kHz	30
	5–10 kHz	20
	10–20 kHz	15
100 Ω Resistor (vs. 10 Ω resistor)	0–2 kHz	20
	2–10 kHz	15
	10–20 kHz	10
Anti-parallel Diodes (vs. 100 Ω resistor)	0–4 kHz	10
	4–10 kHz	6
	10–20 kHz	4

**Table 4:** Summary of final AISAAI suppressor performance (suppressor on vs. off).

Interconnect Cable	Frequency Range	Approximate Interference Reduction (dB)
NBAC (Generic)	0–5 kHz	30
	5–10 kHz	25
	10–20 kHz	15
ASRC (Accuphase)	0–5 kHz	25
	5–10 kHz	20
	10–20 kHz	10

## Data Availability

The data presented in this study are available on request from the corresponding author. The data are not publicly available due to the inclusion of proprietary measurement results.

## Abbreviations

The following abbreviations are used in this manuscript:

AISAAI	Audio Interference Suppressor in Analog Audio Interface
AAIS	Analog Audio Interconnection System
PE	Protective Earth
RLC	Resistance, Inductance, Capacitance
EMI	Electromagnetic Interference
AD_1_	Analog Audio Signal Source Device
AD_2_	Analog Audio Signal Receiver Device
AAI	Analog Audio Interfaces
MPCPE	Connection point of the protective ground of the audio device
PG	Rectifier ground point/Power Ground
PC_1_	Power supply cable for the source (device AD_1_)
PC_2_	Power supply cable for the receiver (device AD_2_)
PEN	Common connection point of the PE conductors of the power distribution network cables
ACPC	Audio Cable Positive Conductor
PL	Parasitic Loop
R_AIS_	Operating resistance within the AISAAI circuit
ACSGC	Audio Cable Signal Ground Conductor
U_OUAD1_	The voltage of the electromotive force of the source AD_1_
Z_OUAD1_	Output impedance of the source (audio device AD_1_)
C_INAD2_	Input (parasitic) capacitance of audio device AD_2_
R_INAD2_	Input resistance of audio device AD_2_
R_ACSG_	Resistance of the (−) negative conductor (Signal Ground) of the unbalanced audio cable
L_ACSG_	Inductance of the (−) negative conductor (Signal Ground) of the unbalanced audio cable
R_ACPC_	Resistance of the (+) positive conductor of the unbalanced audio cable
L_ACPC_	Inductance of the (+) positive conductor of the unbalanced audio cable
C_ACPSG_	Parasitic capacitance of the audio cable between the (+) and (−) conductors
R_PCPE1_	Resistances of the protective earth conductors in power cables PC_1_
R_PCPE2_	Resistances of the protective earth conductors in power cables PC_2_ for supplying AD_1_ and AD_2_
L_PCPE1_	Inductances of the protective earth conductors in power cables PC_1_
L_PCPE2_	Inductances of the protective earth conductors in power cables PC_2_ for supplying AD_1_ and AD_2_
U_PE1_	Voltage of EMI on the protective earth conductors in power cables PC_1_
U_PE2_	Voltage of EMI on the protective earth conductors in power cables PC_2_ for supplying AD_1_ and AD_2_
MPCPE_1_	Connection points of the PE conductor of the power supply cable for the audio devices
MPCPE_2_	Connection points of the PE conductor of the power supply cable for the audio devices
S_AISJ_	Switch that shorts the AISAAI
R_10_	10 Ω resistor with series-connected switch S_10_
R_100_	100 Ω resistor with series-connected switch S_100_
ADC	Antiparallel connection of two diodes, D1 and D2
S_ADC_	Switch for engaging the antiparallel connection of diodes D1 and D2
TVS	Transient Voltage Suppressor
RL	Output load
L/N	Live/Neutral conductors
ASRC	Accuphase Super Refined Cable
NBAC	Unbranded Generic Audio Cable
NB	Non-Branded
Acc	Accuphase
